# Impact of Mn Alloying on Phase Stabilities, Magnetic Properties and Electronic Structures in Fe

**DOI:** 10.3390/ma16206679

**Published:** 2023-10-13

**Authors:** Hao Yang, Jin-Han Yang, Ying Zhao, Han Ma, Yanzhong Tian, Minghui Cai, Shuai Tang, Yandong Liu, Xiang Zhao, Hai-Le Yan, Liang Zuo

**Affiliations:** 1 Key Laboratory for Anisotropy and Texture of Materials (Ministry of Education), School of Material Science and Engineering, Northeastern University, Shenyang 110819, China; 2 Institute of Research of Iron and Steel of Shasteel, Suzhou 215625, China

**Keywords:** Fe, Mn alloying, lattice stability, phase transition, magnetism, first-principles calculations

## Abstract

Impacts of Mn alloying on lattice stabilities, magnetic properties, electronic structures of the bcc and fcc phases and the fcc→bcc phase transition in ${\mathrm{Fe}}_{16-x}{\mathrm{Mn}}_{x}$ (x = 0, 1 and 2) alloys are studied by first-principles calculations. Results show that the doped Mn atom prefers ferromagnetic and antiferromagnetic interaction with the host Fe atoms in the bcc and fcc phases, respectively. In these two phases, the magnetic moment of Mn is smaller and larger than Fe, respectively. The local moment of Fe is decided by the Fe-Mn distance in the bcc phase, whereas in the fcc phase, it is determined by spatial orientation with Mn. In the different phases, Mn prefers different site occupations, which can be understood from the electronic density of states near Fermi energy, implying a possibility of element redistribution during phase transition. The driving force of phase transition decreases with Mn alloying. Both destabilized bcc phase and stabilized fcc phase contribute to the inhibited phase transition, but the latter plays a dominant role. Antiferromagnetism is recognized as the key reason for the enhanced stability of the fcc phase by Mn alloying.

## 1. Introduction

Manganese, the fifth most abundant metal in the Earth’s crust, is a hard, brittle and silvery white metal. In ferroalloys, Mn represents a key alloying element [1,2] owing to its high solid solubility arising from the similar valence electron configurations of Mn ($3d^{5}4s^{2}$) and Fe ($3d^{6}4s^{2}$). In fact, Mn appears in all commercial steels and contributes greatly to strength, ductility, hardenability and high-temperature creep resistance [3,4]. Apart from solid solution strengthening [5,6,7], owing to significant differences of Mn in stabilizing the different phases of ferroalloys [4,8,9], alloying with Mn greatly affects phase transition temperature and thus microstructure phase constituents. By tuning the content of the doped Mn, many advanced steels with an excellent combination of ductility and strength have been developed [10,11]. Furthermore, the prominent role of Mn alloying in tuning phase transition brings several novel functional properties [12], including the shape memory effect and high damping capacity. As is known, the phase stability is intrinsically decided by valence electron concentration, electronic structure, magnetism, lattice volume and so on [13,14,15,16,17,18,19]. Thus, a deep understanding of the impact of Mn alloying on ferroalloys at the level of electronic structure should be helpful for the design of advanced ferroalloys.

To date, the study on the impact of Mn alloying in ferroalloys has focused mainly on magnetism [20,21,22,23,24,25,26,27,28,29,30], elastic properties [31,32,33] and phase transition from face-centered cubic (fcc) to hexagonal close-packed (hcp) [34,35]. Both experiments and theoretical calculations evidence that the FeMn alloy possesses complex magnetic states, which is mainly attributed to the half-filled 3d orbitals of Mn and Fe. The magnetic moments of both Fe and Mn are sensitive to the local chemical environment. With the alloying of Mn, $C_{11}$ and $C_{12}$ of the FeMn alloy are greatly reduced due to the magnetovolume effect, while $C_{44}$ is almost unaffected [32]. For the fcc→hcp transformation, the magnetic ordering is found to play a critical role in the behaviors of phase transition [35]. It is evidenced that the doped Mn and the host Fe prefer to be antiferromagnetically coupled in the fcc phase [36]. The appearance of the antiferromagnetism interaction is found to tend to decrease the stacking fault energy of the fcc phase [37,38], which promotes the occurrence of the fcc→hcp phase transition. Different from the extensive studies on the phase transition from fcc to hcp, despite some reports in pure Fe [39], the impact mechanisms of Mn alloying on the phase transition from the fcc (γ) to the body-centered cubic (bcc, α) phase is less studied. As is known, the fcc→bcc phase transition is vital to the ferroalloy (e.g., steel), since the microstructure modification in this material is mainly achieved by tailoring it. Therefore, clarifying the impact of Mn alloying in the fcc phase, the bcc phase and the fcc→bcc phase transition should be greatly meaningful.

In this work, the impact of Mn alloying on phase stabilities, magnetic properties, electronic structures of the bcc and fcc phases and the phase transition from the fcc to the bcc phase in ${\mathrm{Fe}}_{16-x}{\mathrm{Mn}}_{x}$ (x= 0, 1 and 2) alloys is systematically studied by first-principles calculation. First, the ground-state magnetic and structural preferences in the bcc phase are determined. Herein, due to the complexity of the magnetic states of the FeMn alloy, a two-stage relaxation strategy, i.e., a sole relaxation of the magnetic state followed by a full structural relaxation, is adopted. With the determined structural models, the impacts of Mn alloying on phase stabilities, magnetic properties and electronic structures are investigated (Section 3.1). Second, in analogy to the study on the bcc phase, the magnetic and structural preferences and the impacts of Mn alloying on the fcc phase are studied (Section 3.2). Last, the impact mechanism of Mn doping on the fcc→ bcc phase transition is discussed (Section 3.3).

## 2. Calculational Methods

First-principles density functional theory (DFT) calculations were carried out by using the plane-wave pseudopotential method as implemented in Vienna ab initio Simulation Package (VASP). The Perdew–Burke–Enzerh (PBE) exchange correlation functional in the frame of the generalized gradient approximation (GGA) [40] was used to represent the exchange and correlation energy. The electron–ion interactions were described by the projector augmented wave (PAW) [41] approach. The valence electron configurations of $3d^{6}4s^{2}$ and $3d^{5}4s^{2}$ were utilized for Fe and Mn, respectively. For all calculations, the plane-wave kinetic energy cutoff was set to be 600 eV. The *k*-point mesh with an interval of 0.02 × 2π/Å was used to integrate the Brillouin zone. During structural relaxation, the energy convergence of 10${}^{-5}$ eV and the force convergence of 10${}^{-3}$ eV/Å were adopted [28,30]. For calculations of electronic structure, the Brillouin zone integration was carried out using the tetrahedron method with the Blöchl correction [42]. Three alloys with compositions of ${\mathrm{Fe}}_{16-x}{\mathrm{Mn}}_{x}$ (x= 0, 1 and 2) with a maximum alloying content of 12.5 at.% are investigated. To accurately clarify the impacts of Mn alloying, for all alloys with different doping concentrations, superstructural models with the same atom number (16) are adopted.

## 3. Results and Discussion

### 3.1. Bcc-Structured ${\mathrm{Fe}}_{16-x}{\mathrm{Mn}}_{x}$

#### 3.1.1. Preferred Structural Model

**Figure 1:**
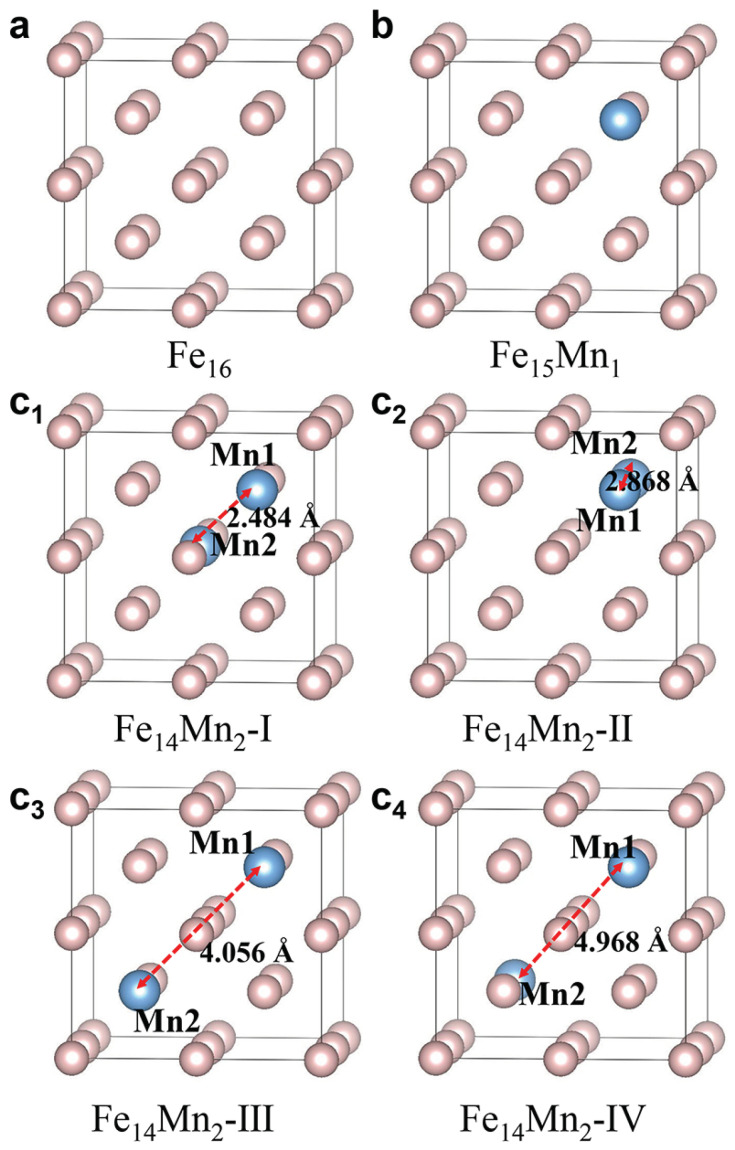
**Bcc superstructural models of ${\mathrm{Fe}}_{16-x}{\mathrm{Mn}}_{x}$ (x= 0, 1 and 2).** (**a**) Pure Fe; (**b**) ${\mathrm{Fe}}_{15}{\mathrm{Mn}}_{1}$; (**c**${}_{1}$–**c**${}_{4}$) Model I to Model IV of ${\mathrm{Fe}}_{14}{\mathrm{Mn}}_{2}$. From Model I to Model IV, $d_{{\mathrm{Mn}}_{1}\text{-}{\mathrm{Mn}}_{2}}$ increases from 2.484 Å to 2.868 Å, 4.056 Å and 4.968 Å.

Figure 1a–c show the 16-atom superstructural models for the bcc phases of pure Fe and the Mn-alloyed ${\mathrm{Fe}}_{15}{\mathrm{Mn}}_{1}$ and ${\mathrm{Fe}}_{14}{\mathrm{Mn}}_{2}$, respectively. For pure Fe, the superstructural model is constituted by stacking eight (2 ×2× 2) bcc unit cells (Figure 1a). The superstructural model of ${\mathrm{Fe}}_{15}{\mathrm{Mn}}_{1}$ is obtained by arbitrarily replacing an Fe atom with Mn in the model of pure Fe, as depicted in Figure 1b. Considering the periodic boundary condition adopted in first-principles calculation, the superstructures in which Mn substitutes different Fe atoms in ${\mathrm{Fe}}_{15}{\mathrm{Mn}}_{1}$ are equivalent. Using a similar method, the structural models of ${\mathrm{Fe}}_{14}{\mathrm{Mn}}_{2}$ are built. Nevertheless, when two Fe atoms are replaced by Mn in ${\mathrm{Fe}}_{14}{\mathrm{Mn}}_{2}$, there exist multiple nonequivalent substitutions. In this work, four superstructural models, designated as Models I to IV, are established. In Models I (Figure 1c${}_{1}$), II (Figure 1c${}_{2}$) and III (Figure 1c${}_{3}$), the two doped Mn atoms (termed as Mn${}_{1}$ and Mn${}_{2}$) are the first (2.484 Å), second (2.868 Å) and third (4.056 Å) nearest neighbors, respectively. In Model IV (Figure 1c${}_{4}$), Mn${}_{1}$ and Mn${}_{2}$ atoms with a distance of 4.968 Å exhibit a symmetric site occupation. From Model I to Model IV, the distance between Mn${}_{1}$ and Mn${}_{2}$ ($d_{{\mathrm{Mn}}_{1}\text{-}{\mathrm{Mn}}_{2}}$) gradually increases.

For the bcc phase of pure Fe, the equilibrium lattice constant $a_{0}^{\mathrm{bcc}}$ and net magnetization $M_{\mathrm{Net}}$ are relaxed to be 2.83 Å and 2.19 µ${}_{B}$/atom, respectively, as listed in Table 1. This is consistent with the values reported in the literature [24,43,44]. In contrast to pure Fe, the determination of the ground-state structural and magnetic configuration in the FeMn alloy is generally not an easy task, since the FeMn alloy typically possesses multiple metastable magnetic states with similar energies [28,29,30,45]. To get out from this dilemma, a two-stage relaxation strategy is adopted in this work for the Mn-doped ${\mathrm{Fe}}_{16-x}{\mathrm{Mn}}_{x}$ alloys. First, the most stable magnetic state is determined by fixing both lattice parameters and atomic positions. Second, with the predetermined magnetic state as the input, a full relaxation is carried out.

**Figure 2:**
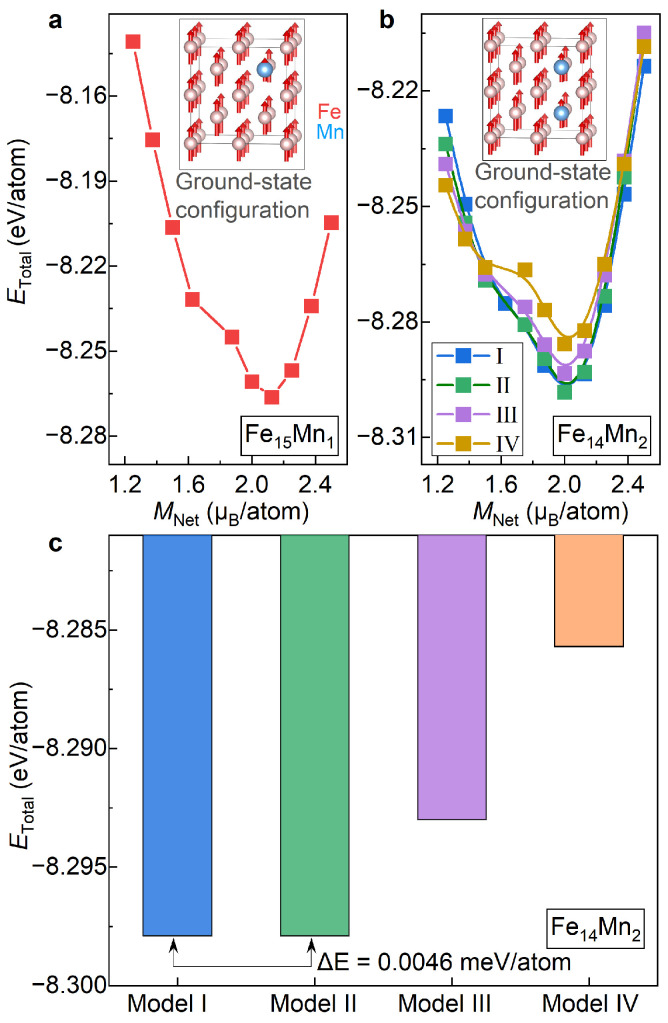
**$E_{\mathrm{Total}}$ of the bcc ${\mathrm{Fe}}_{16-x}{\mathrm{Mn}}_{x}$ (x= 1 and 2) alloys under different magnetic and structural configurations.** (**a**) $E_{\mathrm{Total}}$ vs. $M_{\mathrm{Net}}$ in ${\mathrm{Fe}}_{15}{\mathrm{Mn}}_{1}$; (**b**) $E_{\mathrm{Total}}$ vs. $M_{\mathrm{Net}}$ in ${\mathrm{Fe}}_{14}{\mathrm{Mn}}_{2}$; (**c**) comparison of $E_{\mathrm{Total}}$ of Models I, II, III and IV of ${\mathrm{Fe}}_{14}{\mathrm{Mn}}_{2}$.

Figure 2a presents the total energy ($E_{\mathrm{Total}}$) for the bcc phase of ${\mathrm{Fe}}_{15}{\mathrm{Mn}}_{1}$ plotted as a function of the fixed net magnetization ($M_{\mathrm{Net}}$). Herein, the change in $M_{\mathrm{Net}}$, ranging from 1.25 µ${}_{B}$/atom to 2.5 µ${}_{B}$/atom with an interval of 0.125 µ${}_{B}$/atom, is achieved by using the fixed-spin-moment approach [28]. It is seen that $E_{\mathrm{Total}}$, the bcc phase of ${\mathrm{Fe}}_{15}{\mathrm{Mn}}_{1}$, roughly exhibits a parabolic trend with the increased $M_{\mathrm{Net}}$. The most stable magnetic state of ${\mathrm{Fe}}_{15}{\mathrm{Mn}}_{1}$ is illustrated in the inset of Figure 2a. Clearly, the bcc phase of ${\mathrm{Fe}}_{15}{\mathrm{Mn}}_{1}$ prefers to be ferromagnetic at the ground state. Note that there is a kink around $M_{\mathrm{Net}}$ of 1.7 µ${}_{B}$/atom. This kink, corresponding to a jump in the magnetic moment of the doped Mn, might correspond to a metastable magnetic state.

Figure 2b shows the variation in $E_{\mathrm{Total}}$ for the bcc phase of ${\mathrm{Fe}}_{14}{\mathrm{Mn}}_{2}$ with respect to $M_{\mathrm{Net}}$. Herein, all four site occupations of Mn atoms, i.e., Models I, II, III and IV, are studied. Remarkably, for all four of the examined models, $E_{\mathrm{Total}}$ shows a roughly parabolic tendency against $M_{\mathrm{Net}}$. Like the case of ${\mathrm{Fe}}_{15}{\mathrm{Mn}}_{1}$, for all site occupations of Mn, the most stable magnetic state of the bcc phase of ${\mathrm{Fe}}_{14}{\mathrm{Mn}}_{2}$ is ferromagnetic. The inset of Figure 2b displays the ground-state magnetic configuration of Model II taken as an example. For all the examined four models, $M_{\mathrm{Net}}$ of the most stable magnetic state are around 2.0 µ${}_{B}$/atom, indicating that $M_{\mathrm{Net}}$ of the ground-state magnetism state of ${\mathrm{Fe}}_{14}{\mathrm{Mn}}_{2}$ is independent of the site occupation of Mn. Unlike $M_{\mathrm{Net}}$, the kink in the $E_{\mathrm{Total}}$ vs. $M_{\mathrm{Net}}$ curve, a signal for a possible metastable state, is sensitive to the site occupation of Mn. The kink in Model IV, originating from a reverse of the magnetic moment of the doped Mn, is prominent, while it almost disappears in Models I and II.

With the determined ground-state magnetic states as the input, lattice parameters and atomic positions of ${\mathrm{Fe}}_{15}{\mathrm{Mn}}_{1}$ and ${\mathrm{Fe}}_{14}{\mathrm{Mn}}_{2}$ are fully relaxed, as listed in Table 1. In Figure 2c, $E_{\mathrm{Total}}$ of Models I, II, III and IV of ${\mathrm{Fe}}_{14}{\mathrm{Mn}}_{2}$ are compared. Overall, from Model I to Model IV, i.e., with the increase in $d_{{\mathrm{Mn}}_{1}\text{-}{\mathrm{Mn}}_{2}}$, $E_{\mathrm{Total}}$ of ${\mathrm{Fe}}_{14}{\mathrm{Mn}}_{2}$ shows an increasing trend. Among various models, $E_{\mathrm{Total}}$ values of Models I and II are nearly the same, with a discrepancy of less than 0.01 meV/atom (averaged to all atoms in the superstructure), and exhibit the lowest values. Thus, these two site occupations of Mn are more likely to occur in the bcc phase of ${\mathrm{Fe}}_{14}{\mathrm{Mn}}_{2}$. $E_{\mathrm{Total}}$ values of Models III and IV are higher than Model I/II by 49 meV/atom and 112 meV/atom, respectively. In what follows, for the bcc phase of ${\mathrm{Fe}}_{14}{\mathrm{Mn}}_{2}$, both Models I and II are considered.

#### 3.1.2. Lattice Stabilities and Magnetic Properties

**Figure 3:**
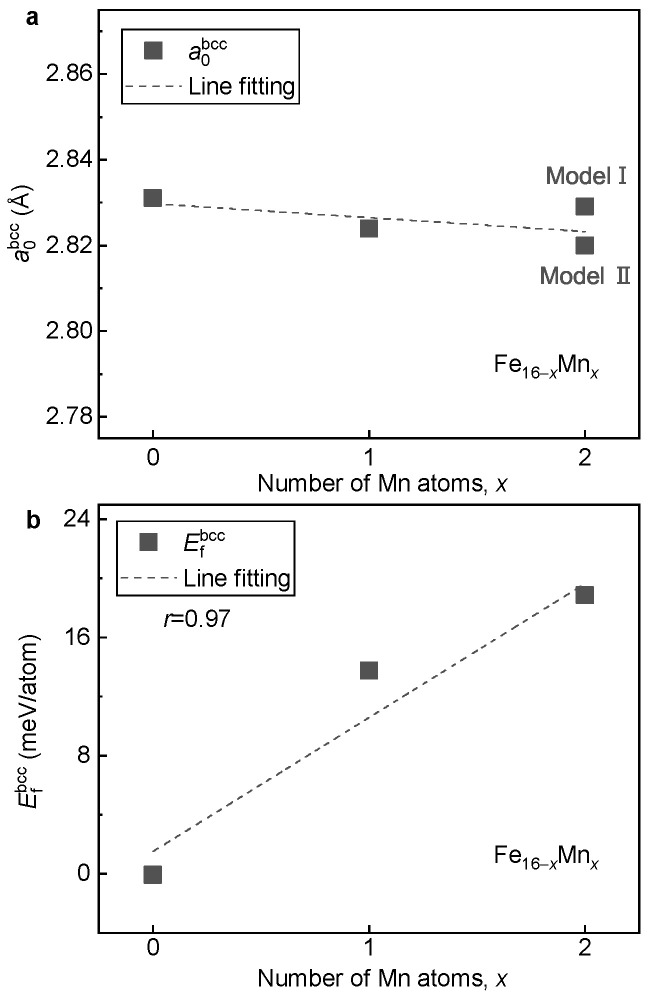
**Dependence of $a_{0}^{\mathrm{bcc}}$ and $E_{f}^{\mathrm{bcc}}$ on the Mn content in the bcc ${\mathrm{Fe}}_{16-x}{\mathrm{Mn}}_{x}$ alloys.** (**a**) $a_{0}^{\mathrm{bcc}}$; (**b**) $E_{f}^{\mathrm{bcc}}$.

With the determined ground-state magnetic and structural models, the effects of Mn alloying on lattice volume, phase stability and magnetic properties of the bcc phase of the ${\mathrm{Fe}}_{16-x}{\mathrm{Mn}}_{x}$ alloys are investigated. Figure 3a shows the equilibrium lattice parameter $a_{0}^{\mathrm{bcc}}$ of the ${\mathrm{Fe}}_{16-x}{\mathrm{Mn}}_{x}$ alloys with respect to the Mn content. It is seen that $a_{0}^{\mathrm{bcc}}$ of the ${\mathrm{Fe}}_{16-x}{\mathrm{Mn}}_{x}$ alloys is insensitive to the Mn content, in good agreement with experimental observation [5]. When two Fe atoms are replaced by Mn in ${\mathrm{Fe}}_{14}{\mathrm{Mn}}_{2}$ with a doping concentration of 12.5 at.%, the variation in $a_{0}^{\mathrm{bcc}}$ is less than 0.01 Å. With the increase in Mn content, even though the magnitude is slight, $a_{0}^{\mathrm{bcc}}$ shows a gradually decreasing tendency.

Figure 3b displays the formation energy $E_{f}^{\mathrm{bcc}}$ of the ${\mathrm{Fe}}_{16-x}{\mathrm{Mn}}_{x}$ alloys with respect to the Mn content. It is seen that with the increase in Mn content, $E_{f}^{\mathrm{bcc}}$ shows a monotonically increasing tendency. This result implies that the alloying of Mn tends to destabilize the bcc phase of the FeMn alloy. With the least square method, a roughly linear relation between $E_{f}^{\mathrm{bcc}}$ and the content of Mn is fitted as follows:

(1) $$E_{f}^{\mathrm{bcc}}=9.1x+1.5$$

where *x* is the doped number of Mn atoms in the 16-atom superstructure. Clearly, when Mn is doped (corresponding to a concentration of 6.25 at.%), the lattice stability of the bcc phase is decreased by around 10 meV/atom.

**Figure 4:**
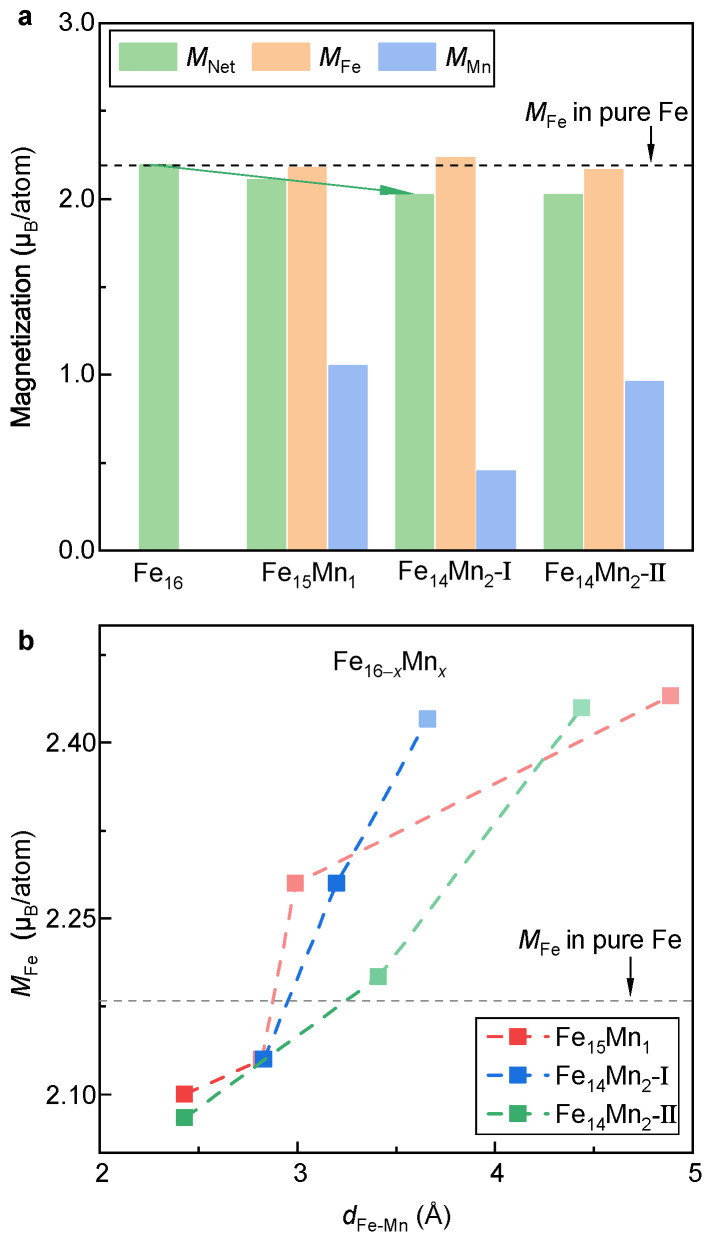
**Net and atom-resolved magnetic moment of the bcc phase.** (**a**) Evolution of magnetization with respect to the doped Mn content. (**b**) Dependence of $M_{\mathrm{Fe}}$ on $d_{\mathrm{Fe}\text{-}\mathrm{Mn}}$.

Figure 4a displays $M_{\mathrm{Net}}$ and atom-resolved magnetic moments of Fe ($M_{\mathrm{Fe}}$) and Mn ($M_{\mathrm{Mn}}$) of the bcc phase of the ${\mathrm{Fe}}_{16-x}{\mathrm{Mn}}_{x}$ alloys. Herein, $M_{\mathrm{Fe}}$ and $M_{\mathrm{Mn}}$ are calculated by taking the mean of atom moments of all Fe and Mn atoms in the supercell, respectively. Clearly, as the Mn content increases, $M_{\mathrm{Net}}$ decreases slightly in a roughly linear manner (indicated in the green arrow). The reduction rate of $M_{\mathrm{Net}}$ is around 0.1 µ${}_{B}$/atom for every introduction of a Mn atom. Notably, $M_{\mathrm{Mn}}$ of the doped Mn atom (0.5–1.0 µ${}_{B}$/atom) is much smaller than $M_{\mathrm{Fe}}$ in pure Fe (~2.2 µ${}_{B}$/atom). In contrast, $M_{\mathrm{Fe}}$ in both ${\mathrm{Fe}}_{15}{\mathrm{Mn}}_{1}$ and ${\mathrm{Fe}}_{14}{\mathrm{Mn}}_{2}$ almost equals that in pure Fe. Thus, the decrease in $M_{\mathrm{Net}}$ with the alloying of Mn should be attributed to a smaller atomic moment of Mn.

Moreover, the decreased $M_{\mathrm{Net}}$ gives us a clue to understand the shortened $a_{0}^{\mathrm{bcc}}$ with the alloying of Mn. The atom radius of Mn (161 pm) is slightly larger than that of Fe (156 pm) [46]. It is thus expected naively that there would be an increase in the lattice constant with the alloying of Mn, which is opposite to the experimental observation (Figure 3a). Thus, there should be an extra mechanism deciding lattice volume in the ${\mathrm{Fe}}_{16-x}{\mathrm{Mn}}_{x}$ (x = 0, 1 and 2) alloys. Apart from atom radius, lattice volume is sensitive to the magnetic moment, i.e., the magnetovolume effect [47]. A decrease in magnetic moment generally tends to cause a shrinkage of lattice volume. Thus, the decreased $M_{\mathrm{Net}}$ could be responsible for the shortened lattice constant. To verify this, the spin non-polarized calculations are carried out. $a_{0}^{\mathrm{bcc}}$ values of Fe, ${\mathrm{Fe}}_{15}{\mathrm{Mn}}_{1}$ and ${\mathrm{Fe}}_{14}{\mathrm{Mn}}_{2}$ in the non-magnetic state are determined to be 2.756, 2.761 and 2.770 Å, respectively. Clearly, an opposite trend, i.e., an increase in the lattice constant with the alloying of Mn, is obtained. This result verifies the key role of magnetism in the lattice volume of the bcc phase of the ${\mathrm{Fe}}_{16-x}{\mathrm{Mn}}_{x}$ (x = 0, 1 and 2) alloys.

Note that despite the similar $M_{\mathrm{Net}}$ of Models I and II of ${\mathrm{Fe}}_{14}{\mathrm{Mn}}_{2}$, $M_{\mathrm{Fe}}$ and $M_{\mathrm{Mn}}$ in these two models are obviously distinct. It suggests that the site occupation of Mn would greatly affect the local magnetic moments of Fe and Mn, aligning well with the previous investigations [29]. To make it clear, the relation between the moment of Fe and the distance between Fe and Mn (i.e., $d_{\mathrm{Fe}\text{-}\mathrm{Mn}}$) is investigated, as shown in Figure 4b. For ${\mathrm{Fe}}_{14}{\mathrm{Mn}}_{2}$, the Fe-Mn distance is determined by averaging the distances of the Fe atom from the Mn${}_{1}$ and Mn${}_{2}$ atoms. As a reference, $M_{\mathrm{Fe}}$ in pure Fe is plotted in the dashed line. Roughly speaking, $M_{\mathrm{Fe}}$ is positively correlated to $d_{\mathrm{Fe}\text{-}\mathrm{Mn}}$. When $d_{\mathrm{Fe}\text{-}\mathrm{Mn}}$ is less than ~3 Å, $M_{\mathrm{Fe}}$ of the FeMn alloy is smaller than that of pure Fe. Unexpectedly, when $d_{\mathrm{Fe}\text{-}\mathrm{Mn}}$ > 3 Å, $M_{\mathrm{Fe}}$ is higher than that of pure Fe. Compared with Model II, the sensitivity of $M_{\mathrm{Fe}}$ against $d_{\mathrm{Fe}\text{-}\mathrm{Mn}}$ in Model I is relatively stronger. At a $d_{\mathrm{Fe}\text{-}\mathrm{Mn}}$ of ~3.6 Å, $M_{\mathrm{Fe}}$ equals 2.42 $\mu_{B}/$atom, which is larger than that of pure Fe. This may explain the slightly larger mean $M_{\mathrm{Fe}}$ in Model I compared to pure Fe (Figure 4a).

#### 3.1.3. Electronic Structures

**Figure 5:**
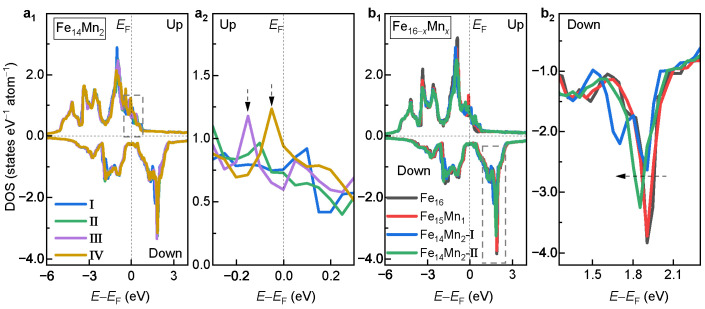
**DOS of the bcc phase.** (**a**${}_{1}$) Comparison of DOSs of Models I, II, III and IV of ${\mathrm{Fe}}_{14}{\mathrm{Mn}}_{2}$. (**a**${}_{2}$) Enlarged view of the dashed box in **a**${}_{1}$. (**b**${}_{1}$) Dependence of DOS on the doped Mn content. (**b**${}_{2}$) Enlarged view of the dashed box in **b**${}_{1}$.

To understand the site occupation preference and magnetic properties, the electronic density of states (DOSs) of the bcc phase of the ${\mathrm{Fe}}_{16-x}{\mathrm{Mn}}_{x}$ alloys was investigated. Figure 5a${}_{1}$ displays the DOSs of Models I, II, III and IV of ${\mathrm{Fe}}_{14}{\mathrm{Mn}}_{2}$. Overall, the DOS curves of the examined four models with different site occupations of Mn exhibit similar features. A slight difference appears in the majority-spin channel near $E_{F}$, as shown (enlarged) in Figure 5a${}_{2}$. Clearly, for both Models III and IV, there exists a prominent state peak near $E_{F}$ (0~−0.2 eV), indicated in vertical arrows. Nevertheless, both Models I and II exhibit a much flatter DOS structure near $E_{F}$. As is known, the DOS structure near $E_{F}$ strongly affects the lattice stability [14,48]. Generally, the smaller the DOS near $E_{F}$, the more stable the lattice. Thus, the fewer states near $E_{F}$ in Models I and II may explain their energy advantages.

Figure 5b${}_{1}$ compares the DOSs for the bcc phases of the ${\mathrm{Fe}}_{16-x}{\mathrm{Mn}}_{x}$ (x= 0, 1 and 2) alloys with different Mn contents. For ${\mathrm{Fe}}_{14}{\mathrm{Mn}}_{2}$, the DOSs of both Models I and II are plotted. Roughly speaking, it is seen that the DOSs of the bcc phase of different ${\mathrm{Fe}}_{16-x}{\mathrm{Mn}}_{x}$ alloys are nearly overlapping. The reason could be twofold. First, the difference in valence electron number between the doped Mn and the host Fe is small (1). Second, the doping amount of Mn studied in this work is not huge. Further analyses show that with the increased Mn content, the minority-spin DOS slightly moves towards lower energy, as indicated by the dashed arrow in Figure 5b${}_{2}$. The left movement of the minority-spin DOS decreases the asymmetry with the majority-spin DOS, i.e., magnetic exchange splitting, bringing about a decrease in net magnetization [44]. This is in good agreement with the result of the magnetic properties in Figure 4a.

### 3.2. Fcc-Structured ${\mathrm{Fe}}_{16-x}{\mathrm{Mn}}_{x}$

#### 3.2.1. Preferred Structural Models

**Figure 6:**
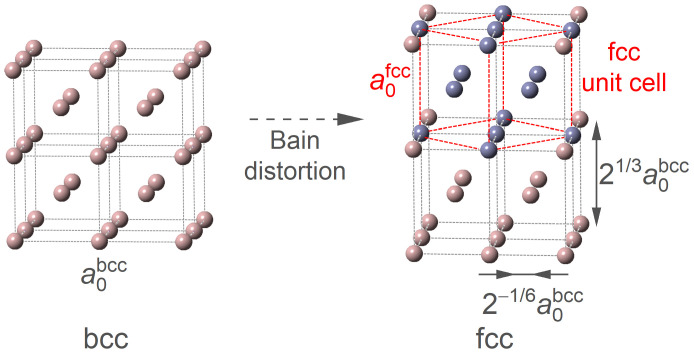
**Illustration of obtaining the fcc model from the bcc one by Bain distortion.**

We now shift our attention from the bcc phase to the fcc phase. Herein, to keep the consistency of site occupation of the doped Mn atoms, the superstructures of the fcc phase are obtained by applying the Bain strain on the models of the bcc phase, as illustrated in Figure 6. During the distortion, the *a* and *b* axes of the bcc phase are shrunk by a normal strain of $2^{-1/6}$, while the *c* axis is extended with a strain of $2^{1/3}$. As indicated by the red dashed lattice, there clearly exists a normal fcc unit cell in the distorted bcc superstructure.

**Figure 7:**
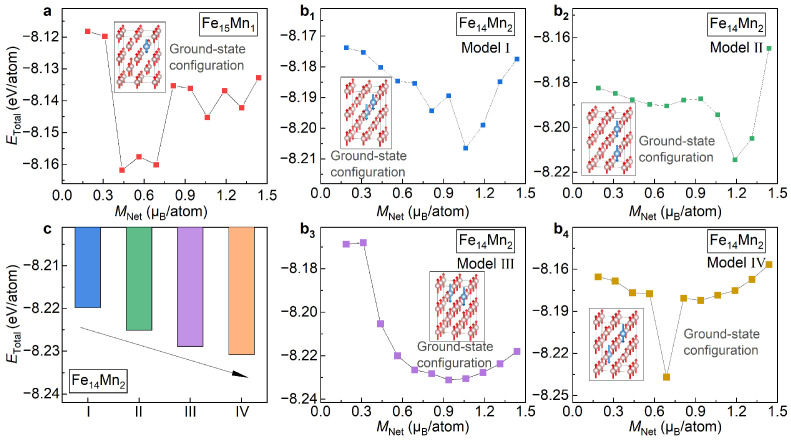
**$E_{\mathrm{Total}}$ of the fcc phase under different magnetic and structural configurations.** (**a**) $E_{\mathrm{Total}}$ vs. $M_{\mathrm{Net}}$ of ${\mathrm{Fe}}_{15}{\mathrm{Mn}}_{1}$; (**b**${}_{1}$–**b**${}_{4}$) $E_{\mathrm{Total}}$ vs. $M_{\mathrm{Net}}$ of Models I, II, III and IV of ${\mathrm{Fe}}_{14}{\mathrm{Mn}}_{2}$. The insets in (**a**,**b**) illustrate the optimized ground-state configurations. The pink and light blue balls represent Fe and Mn atoms, respectively. (**c**) Comparison of $E_{\mathrm{Total}}$ of Models I, II, III and IV of ${\mathrm{Fe}}_{14}{\mathrm{Mn}}_{2}$.

Starting from the models built by Bain distortion of the fcc phase, the structural relaxations are performed to obtain the ground-state structure and magnetism configuration. In analogy to the study in the bcc phase, a two-stage relaxation strategy, i.e., a sole relaxation of the magnetic state followed by a full relaxation, is adopted. At the relaxation of the magnetic state, considering that $M_{\mathrm{Net}}$ of the fcc Fe is around 1.0 µ${}_{B}$/atom, $M_{\mathrm{Net}}$ scans from 0.1 µ${}_{B}$/atom to 1.5 µ${}_{B}$/atom with an interval of 0.125 µ${}_{B}$/atom. Figure 7a displays $E_{\mathrm{Total}}$ of ${\mathrm{Fe}}_{15}{\mathrm{Mn}}_{1}$ with respect to the fixed $M_{\mathrm{Net}}$. It is seen that the most stable magnetic state for the fcc phase of ${\mathrm{Fe}}_{15}{\mathrm{Mn}}_{1}$ appears at $M_{\mathrm{Net}}$ of ~0.5 µ${}_{B}$/atom. As shown in the inset of Figure 7a, under this magnetic state, the magnetic moment of the doped Mn and the host Fe atoms are antiparallelly arranged, i.e., the antiferromagnetic interaction. This is greatly different from the magnetic state in the bcc phase where the moments of the Mn and Fe atoms are ferromagnetically coupled. Figure 7b${}_{1}$–b${}_{4}$ shows the dependence of $E_{\mathrm{Total}}$ on $M_{\mathrm{Net}}$ of ${\mathrm{Fe}}_{14}{\mathrm{Mn}}_{2}$ for Models I, II, III and IV, respectively. The inset in each figure shows the most stable magnetic state. Remarkably, in all models, despite the different $M_{\mathrm{Net}}$, the magnetic moments of the doped Mn atoms and the host Fe atoms are antiferromagnetically coupled.

Based on the optimized most stable magnetic state as the input, the structural models of ${\mathrm{Fe}}_{15}{\mathrm{Mn}}_{1}$ and ${\mathrm{Fe}}_{14}{\mathrm{Mn}}_{2}$, including the lattice parameter and atomic position and magnetic moment, are fully relaxed, as listed in Table 2. In Figure 7c, $E_{\mathrm{Total}}$ of the relaxed four structural models of ${\mathrm{Fe}}_{14}{\mathrm{Mn}}_{2}$ are compared. From Model I to Model IV, i.e., with the increased $d_{{\mathrm{Mn}}_{1}\text{-}{\mathrm{Mn}}_{2}}$, $E_{\mathrm{Total}}$ shows a monotonically decreasing tendency. This is completely opposite to the trend observed in the bcc lattice (Figure 2c), where $E_{\mathrm{Total}}$ gradually elevates with the increase in $d_{{\mathrm{Mn}}_{1}\text{-}{\mathrm{Mn}}_{2}}$ (indicated by the black arrow). The different site occupation preference of Mn in the fcc and bcc phases implies that during the fcc→bcc phase transition, the redistribution of Mn in the Fe matrix would happen. This is in good agreement with experimental observations [1,2]. Among various models, Model IV possesses the lowest energy. Models III, II and I have energies higher than Model IV by 19, 57 and 110 meV/atom, respectively. In what follows, the site occupation of Model IV is considered for ${\mathrm{Fe}}_{14}{\mathrm{Mn}}_{2}$.

#### 3.2.2. Lattice Stabilities and Magnetic Properties

**Figure 8:**
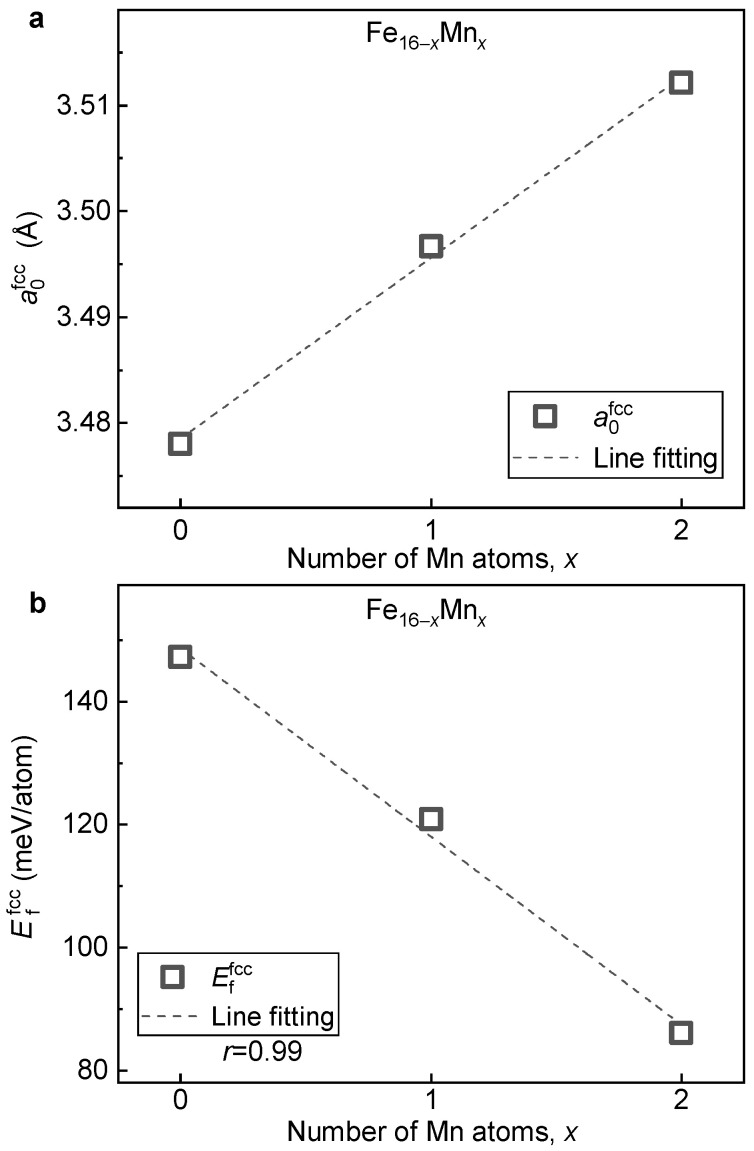
**Dependence of $a_{0}^{\mathrm{fcc}}$ and $E_{f}^{\mathrm{fcc}}$ on the Mn content in the fcc ${\mathrm{Fe}}_{16-x}{\mathrm{Mn}}_{x}$ alloys.** (**a**) $a_{0}^{\mathrm{fcc}}$; (**b**) $E_{f}^{\mathrm{fcc}}$.

With the relaxed ground-state structure, the impact of Mn content on lattice volume, phase stability and magnetic properties of the fcc phase of the ${\mathrm{Fe}}_{16-x}{\mathrm{Mn}}_{x}$ (x= 0, 1 and 2) alloys is investigated. Figure 8a presents the lattice constant ($a_{0}^{\mathrm{fcc}}$) of the fcc phase of the ${\mathrm{Fe}}_{16-x}{\mathrm{Mn}}_{x}$ alloys plotted as a function of Mn content. With the increase in Mn content, $a_{0}^{\mathrm{fcc}}$ shows a linearly increasing tendency. This change, opposite to what is observed in the bcc structure (Figure 3a), is in agreement with the previous theoretical calculation [34] and experimental measurement [5]. The expanded lattice with Mn alloying could be attributed to the fact that the atom radius of Mn (161 pm) is slightly larger than that of Fe (156 pm) [46]. Note that when two Mn atoms are doped in the 16-atom superstructural model, i.e., from pure Fe to ${\mathrm{Fe}}_{14}{\mathrm{Mn}}_{2}$, $a_{0}^{\mathrm{fcc}}$ increases by a value of larger than 0.03 Å. This greatly differs from that of the bcc phase where the variation in $a_{0}^{\mathrm{bcc}}$ is less than 0.01 Å under the same doping concentration. Thus, compared to the bcc phase, $a_{0}^{\mathrm{fcc}}$ is more sensitive to the alloying of Mn.

Figure 8b displays the formation energy of the fcc phase ($E_{f}^{\mathrm{fcc}}$) of the ${\mathrm{Fe}}_{16-x}{\mathrm{Mn}}_{x}$ alloys plotted as a function of the Mn content. With the increase in Mn content, $E_{f}^{\mathrm{fcc}}$ shows a monotonically decreasing tendency. Thus, the alloying of Mn tends to stabilize the fcc phase, which is in contrast to the destabilized effect on the bcc phase (Figure 3b). By the least square method, a linear relation between $E_{f}^{\mathrm{fcc}}$ and the doped Mn content is fitted as follows:

(2) $$E_{f}^{\mathrm{fcc}}=-30.6x+148.7$$

where *x* is the doped number of Mn in the 16-atom supercell. From Equation (2), it is clear that when a Mn atom is doped, the lattice stability of the fcc phase increases by a value of around −30 meV/atom. Note that apart from the opposite sign, the variation in formation energy caused by Mn alloying in the fcc phase is around three times larger than that of the bcc phase.

**Figure 9:**
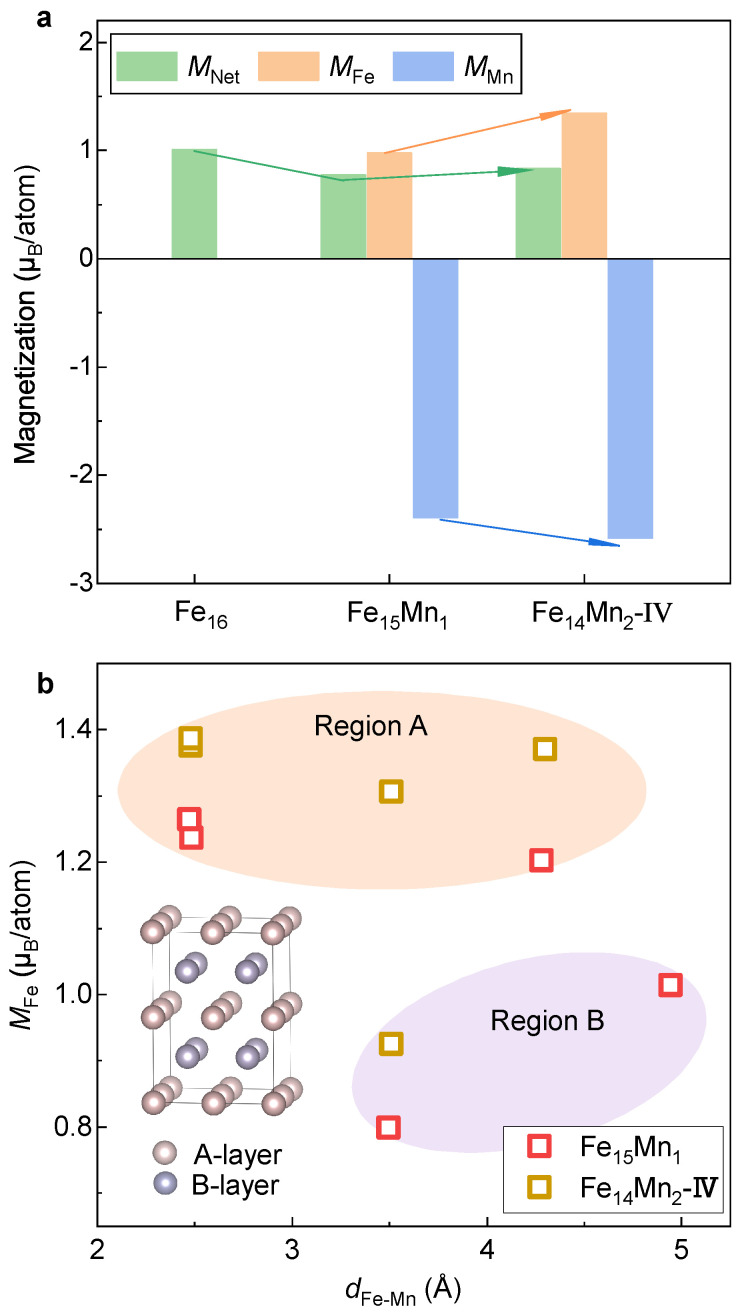
**Net and atom-resolved magnetic moment of the fcc phase.** (**a**) Evolution of magnetization with respect to the doped Mn content. (**b**) Dependence of $M_{\mathrm{Fe}}$ on $d_{\mathrm{Fe}\text{-}\mathrm{Mn}}$.

Figure 9a displays $M_{\mathrm{Net}}$ and atom-resolved $M_{\mathrm{Mn}}$ and $M_{\mathrm{Fe}}$ in the fcc phase of the ${\mathrm{Fe}}_{16-x}{\mathrm{Mn}}_{x}$ (x= 0, 1 and 2) alloys. Clearly, the signs of $M_{\mathrm{Mn}}$ and $M_{\mathrm{Fe}}$ are opposite, indicating an antiferromagnetic interaction between Fe and Mn. In analogy to the bcc phase, with the alloying of Mn, $M_{\mathrm{Net}}$ of the fcc phase decreases from 1.02 µ${}_{B}$/atom in pure Fe to 0.77 µ${}_{B}$/atom in ${\mathrm{Fe}}_{15}{\mathrm{Mn}}_{1}$. However, different from the case in the bcc phase where the reduced $M_{\mathrm{Net}}$ is attributed to smaller $M_{\mathrm{Mn}}$ (0.5–1.0 µ${}_{B}$/atom) compared with $M_{\mathrm{Fe}}$ (~2.2 µ${}_{B}$/atom), the decrease in $M_{\mathrm{Net}}$ arises from antiferromagnetic interactions between Fe and Mn in the fcc phase. Surprisingly, opposite to the case in the bcc phase that $M_{\mathrm{Mn}}$ is around a half of $M_{\mathrm{Fe}}$, $M_{\mathrm{Mn}}$ in the fcc phase (~−2.4 µ${}_{B}$/atom) is about two times larger than that of $M_{\mathrm{Fe}}$ (~1.0 µ${}_{B}$/atom) despite a reversed direction of moment. Furthermore, in contrast to a monotonically decreasing tendency of $M_{\mathrm{Net}}$ with the Mn doping in the bcc phase, $M_{\mathrm{Net}}$ of ${\mathrm{Fe}}_{14}{\mathrm{Mn}}_{2}$ in the fcc phase is slightly larger than that of ${\mathrm{Fe}}_{15}{\mathrm{Mn}}_{1}$ by 0.06 µ${}_{B}$/atom. Apart from $M_{\mathrm{Net}}$, both $M_{\mathrm{Fe}}$ and $M_{\mathrm{Mn}}$ in ${\mathrm{Fe}}_{14}{\mathrm{Mn}}_{2}$ are larger than those in ${\mathrm{Fe}}_{15}{\mathrm{Mn}}_{1}$, as highlighted by the arrows in Figure 9a.

To clarify the impact of Mn doping on the magnetic moment of Fe, $M_{\mathrm{Fe}}$ values of different Fe atoms in ${\mathrm{Fe}}_{15}{\mathrm{Mn}}_{1}$ and ${\mathrm{Fe}}_{14}{\mathrm{Mn}}_{2}$ alloys are plotted in Figure 9b. Unlike the bcc phase where $M_{\mathrm{Fe}}$ is roughly positively correlated to $d_{\mathrm{Fe}\text{-}\mathrm{Mn}}$, no clear pattern is seen between $M_{\mathrm{Fe}}$ and $d_{\mathrm{Fe}\text{-}\mathrm{Mn}}$ for the fcc phase. In contrast, in terms of the magnitude of $M_{\mathrm{Fe}}$, the Fe atoms in the fcc FeMn alloys can be clearly classified into two categories. One is centered around 1.3 µ${}_{B}$/atom (termed as Region A), while the other is in the range of 0.8–1.0 µ${}_{B}$/atom (Region B). Amazingly, it is found that the Fe atoms located in Regions A and B come from the layers A and B in the structural model inserted in Figure 9b. The key difference between layers A and B is that the antiferromagnetically coupled Mn atom is located in the B layer and there is no Mn in layer A. Note that the two Mn atoms in ${\mathrm{Fe}}_{14}{\mathrm{Mn}}_{2}$ are located in the two B layers, respectively, while only one B layer has Mn in ${\mathrm{Fe}}_{15}{\mathrm{Mn}}_{1}$. This result shows that different from the $d_{\mathrm{Fe}\text{-}\mathrm{Mn}}$ domination in the bcc phase, the local moment of Fe is heavily affected by the spatial orientation of its position with respect to the doped Mn.

#### 3.2.3. Electronic Structures

**Figure 10:**
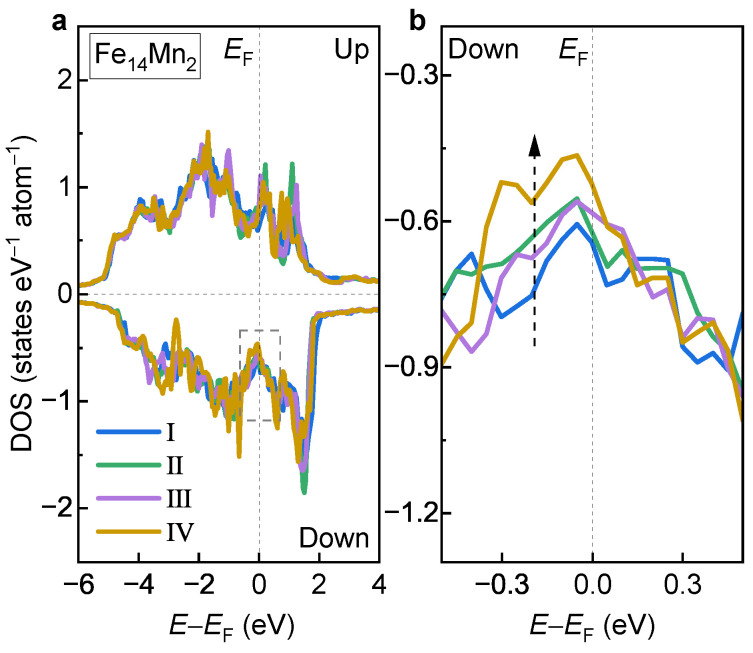
**(a) DOS of Models I, II, III and IV of the fcc ${\mathrm{Fe}}_{14}{\mathrm{Mn}}_{2}$ alloy.** (**b**) Enlarged view of the dashed box in (**a**).

To understand the site occupation preferences of the doped Mn in the fcc phase, the DOSs of Models I, II, III and IV of the fcc ${\mathrm{Fe}}_{14}{\mathrm{Mn}}_{2}$ alloy are compared in Figure 10a. Overall, the DOS curves of the examined four models show similar features. Detailed analyses show that there exists an obvious difference in the minority-spin DOS near $E_{F}$, as shown (enlarged) in Figure 10b. Clearly, from Model I to Model IV, the state amount near $E_{F}$ shows a monotonically decreasing tendency, as indicated by the dashed arrow. This is in excellent agreement with the order of the decreased $E_{\mathrm{Total}}$ in Figure 7c. Notably, Model IV possesses the fewest electron states near $E_{F}$, which corresponds well with its highest phase stability.

### 3.3. Phase Transition

We now shift our attention from the single bcc/fcc phase to the fcc→bcc phase transition. First, the stabilities of the fcc and the bcc phases are compared and the underlying mechanism in electronic structure is discussed. Second, the impact of the Mn doping on the fcc→bcc phase transition is investigated. Third, the key factors dominating the adjustment of Mn alloying on the fcc→bcc phase transition are discussed.

#### 3.3.1. Phase Transition from fcc to bcc

Table 1 and Table 2 list $E_{\mathrm{Total}}$ of the ${\mathrm{Fe}}_{16-x}{\mathrm{Mn}}_{x}$ alloys for the bcc and the fcc phase, respectively. For both pure Fe and various Mn-doped FeMn alloys, $E_{\mathrm{Total}}$ of the bcc phase is obviously smaller than that of the corresponding fcc phase. For instance, $E_{\mathrm{Total}}$ of ${\mathrm{Fe}}_{15}{\mathrm{Mn}}_{1}$ at the bcc phase is −8.266 eV/atom, which is smaller than that at the fcc phase (−8.159 eV/atom). Thus, compared with the fcc phase, the bcc phase exhibits a higher phase stability. This is in good agreement with the previous calculations [39,50] and experimental observation [51] that a phase transition from the fcc phase to the ferromagnetic bcc phase occurs in the ferroalloys. In fact, the rich microstructure of steel materials is indeed attributed to the existence of fcc→bcc phase transition.

**Figure 11:**
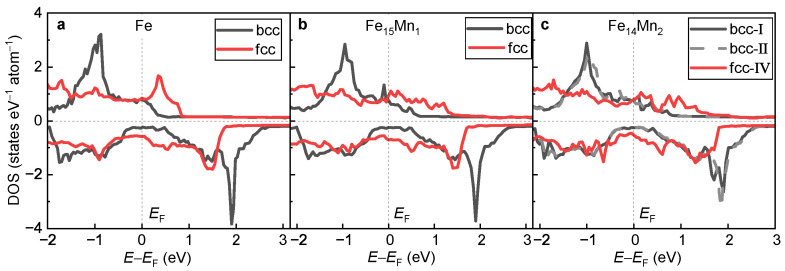
**Comparison of DOSs of the bcc and fcc phases.** (**a**) Pure Fe; (**b**) ${\mathrm{Fe}}_{15}{\mathrm{Mn}}_{1}$; (**c**) ${\mathrm{Fe}}_{14}{\mathrm{Mn}}_{2}$.

In Figure 11a–c, the DOSs of the fcc and the bcc phases for pure Fe, ${\mathrm{Fe}}_{15}{\mathrm{Mn}}_{1}$ and ${\mathrm{Fe}}_{14}{\mathrm{Mn}}_{2}$ are compared, respectively. For all alloys, the minority-spin DOS near $E_{F}$ of the bcc phase is obviously less than that of the fcc phase. The greater the amount of DOS near $E_{F}$, the weaker the phase stability [14,48]. This explains, in view of electronic structure, the phase transition from the high-energy fcc to the low-energy bcc phase. Furthermore, for all alloys, the exchange splitting of the DOS structure in the bcc phase is obviously greater than that of the fcc phase, which is consistent with the larger $M_{\mathrm{Net}}$ of the bcc phase.

#### 3.3.2. Impact of Mn on Phase Transition

The discrepancy of favorable structural models of the bcc and the fcc phase in ${\mathrm{Fe}}_{14}{\mathrm{Mn}}_{2}$ implies a possibility of Mn redistribution during the fcc→bcc phase transition. We now focus on its impact on the behavior of phase transition. To make it clear, by using two different approaches, the energy difference $\Delta E_{\mathrm{fcc}\to \mathrm{bcc}}$ between the fcc and the bcc phases defined as $E_{\mathrm{bcc}}-E_{\mathrm{fcc}}$, i.e., the driving force for the fcc→bcc phase transition, is calculated. First, $\Delta E_{\mathrm{fcc}\to \mathrm{bcc}}$ is computed by adopting the most stable structural models for both the fcc (Model IV) and the bcc (Model I/II) phases to represent the case with Mn diffusion during phase transition. For a better illustration, $\Delta E_{\mathrm{fcc}\to \mathrm{bcc}}$ calculated in this way is termed as $\Delta E_{\mathrm{fcc}\to \mathrm{bcc}}^{0}$. Second, $\Delta E_{\mathrm{fcc}\to \mathrm{bcc}}$ is recalculated by adopting the fcc and bcc phases with the same site occupation of Mn to mimic the case without Mn diffusion. Herein, $\Delta E_{\mathrm{fcc}\to \mathrm{bcc}}$ values calculated for Models I, II, III and IV are designated as $\Delta E_{\mathrm{fcc}\to \mathrm{bcc}}^{I}$, $\Delta E_{\mathrm{fcc}\to \mathrm{bcc}}^{\mathrm{II}}$, $\Delta E_{\mathrm{fcc}\to \mathrm{bcc}}^{\mathrm{III}}$ and $\Delta E_{\mathrm{fcc}\to \mathrm{bcc}}^{\mathrm{IV}}$, respectively.

**Figure 12:**
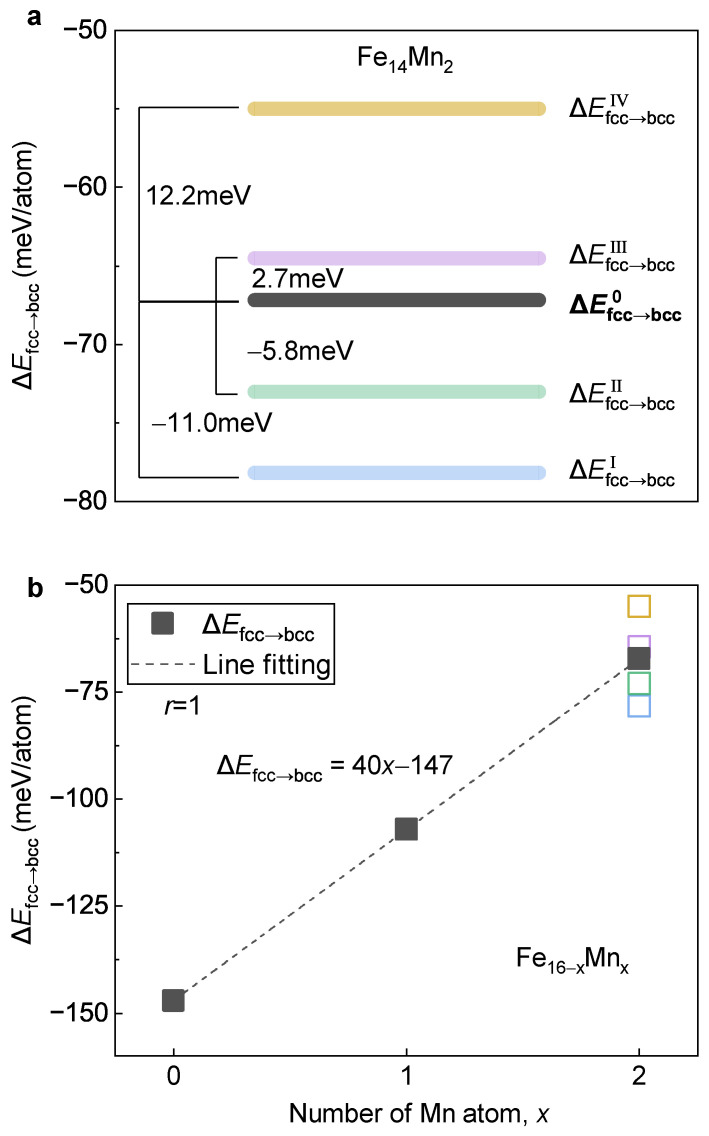
**Variation of $\Delta E_{\mathrm{fcc}\to \mathrm{bcc}}$ against the Mn content.** (**a**) $\Delta E_{\mathrm{fcc}\to \mathrm{bcc}}$ of ${\mathrm{Fe}}_{14}{\mathrm{Mn}}_{2}$ with different site occupations of Mn. (**b**) Dependence of $\Delta E_{\mathrm{fcc}\to \mathrm{bcc}}$ on Mn content. The blocks in a and the open squares in (**b**) with various colors represent different occupation models of ${\mathrm{Fe}}_{14}{\mathrm{Mn}}_{2}$.

Figure 12a displays the determined $\Delta E_{\mathrm{fcc}\to \mathrm{bcc}}$ of ${\mathrm{Fe}}_{14}{\mathrm{Mn}}_{2}$. Despite the different values of $\Delta E_{\mathrm{fcc}\to \mathrm{bcc}}^{I}$ to $\Delta E_{\mathrm{fcc}\to \mathrm{bcc}}^{\mathrm{IV}}$, they are clustered around $\Delta E_{\mathrm{fcc}\to \mathrm{bcc}}^{0}$ with a deviation at the level of ±10 meV/atom. From Model IV to Model I, the absolute value of the energy difference between the fcc and the bcc phases gradually increases, implying an increase in the driving force of the fcc→bcc phase transition. It is revealed that the high-temperature fcc phase prefers Model IV, while the low-temperature bcc phase favors Model I/II. Thus, during the phase transition from the fcc to the bcc phase, the Mn atoms prefer a diffusion from the site occupation in Model IV to that in Model I/II. Accompanied by this diffusion, the driving force of the phase transition increases with a value of ~12 meV/atom (calculated by $\Delta E_{\mathrm{fcc}\to \mathrm{bcc}}^{0}-\Delta E_{\mathrm{fcc}\to \mathrm{bcc}}^{\mathrm{IV}}$).

Figure 12b presents $\Delta E_{\mathrm{fcc}\to \mathrm{bcc}}$ of the ${\mathrm{Fe}}_{16-x}{\mathrm{Mn}}_{x}$ (x= 0, 1 and 2) alloys plotted as a function of Mn content. For ${\mathrm{Fe}}_{14}{\mathrm{Mn}}_{2}$, $\Delta E_{\mathrm{fcc}\to \mathrm{bcc}}^{0}$ is displayed and the deviations of $\Delta E_{\mathrm{fcc}\to \mathrm{bcc}}^{I}$ to $\Delta E_{\mathrm{fcc}\to \mathrm{bcc}}^{\mathrm{IV}}$ are plotted in open symbols. Clearly, with the increase in Mn content, $\Delta E_{\mathrm{fcc}\to \mathrm{bcc}}$ shows a perfectly linear increasing tendency with a Pearson coefficient of near 1. An increase in $\Delta E_{\mathrm{fcc}\to \mathrm{bcc}}$ suggests a decrease in driving force for the fcc→bcc phase transition, corresponding to a lowered phase transition temperature. In other words, using a more colloquial expression, Mn tends to stabilize the high-temperature fcc phase (austenite) against the low-temperature bcc phase (e.g., ferrite), which is in good agreement with the experimental observations [52]. With the least square method, a linear relation between $\Delta E_{\mathrm{fcc}\to \mathrm{bcc}}$ and the content of Mn is obtained, as follows:

(3) $$\Delta E_{\mathrm{fcc}\to \mathrm{bcc}}=40x-147$$

where *x* represents the substituted number of Mn atoms in the 16-atom superstructure.

#### 3.3.3. Key Factor Deciding Mn Tailored Phase Transition

In terms of lattice stability, the alloying of Mn tends to destabilize the bcc phase (Figure 3b) and stabilize the fcc phase (Figure 8b). Clearly, both effects of Mn alloying on the bcc phase and the fcc phase have the same sign as its impact on the fcc→bcc phase transition, i.e., decreasing the driving force from fcc to bcc. Thus, for Mn alloying in ferroalloys, both the destabilizing effect on the bcc phase and the stabilizing effect on the fcc phase contribute to the inhibited fcc→bcc phase transition. Note that when one more Mn atom is doped, as seen in Equation (3), the driving force of the fcc→bcc phase transition ($\Delta E_{\mathrm{fcc}\to \mathrm{bcc}}$) increases to 40 meV/atom. According to Equations (1) and (2), it is evident that the destabilized effect on the bcc phase contributes ~10 meV, while the stabilized effect on the fcc phase contributes ~30 meV/atom. Clearly, the contribution of the stabilizing effect on the fcc phase is around three times larger than that of the destabilizing effect on the bcc phase. Therefore, the influence of Mn doping on the stability of the fcc phase, i.e., the strongly stabilizing effect, should dominate the inhibited role in the fcc→bcc phase transition.

Lastly, the mechanism behind the stabilizing effect of Mn in the fcc phase is discussed. As is known, valence electron concentration (VEC), lattice volume and magnetism are the three key factors deciding lattice stability [13,14,15]. When Mn replaces Fe, the VEC is decreased, owing to fewer valence electrons of Mn (7) against Fe (8). Generally, the lower the VEC, the weaker the stability of the fcc phase [15]. Thus, VEC is not the reason for the improved stability of the fcc phase. For lattice volume, it increases monotonically with the alloying of Mn. It is known that the expansion of lattice volume tends to weaken bonding strength and thus it also might not be the reason for the improved lattice stability of the fcc phase. As for magnetism, it is revealed that the doped Mn and the host Fe prefer to be antiferromagnetically coupled and the interaction strength is gradually enhanced with the increased doping concentration. It is well-recognized that antiferromagnetism generally strongly stabilizes the fcc structure [34,35]. Thus, antiferromagnetism could be responsible for the stabilizing effect of the fcc phase in Mn doping and further for the inhibition of the fcc→bcc phase transition.

## 4. Conclusions

The impact of Mn alloying on magnetic and structural preferences, lattice stabilities, magnetic properties and electronic structures of the bcc and the fcc phases and the fcc→bcc phase transition is studied systematically by first-principles calculations. The main findings are as follows.

(i) Mn prefers ferromagnetic and antiferromagnetic interaction with Fe in the bcc and fcc phases, respectively. In these two phases, the magnetic moment of Mn is smaller and larger than Fe, respectively. The local moment of Fe is decided by the Fe-Mn distance in the bcc phase, whereas in the fcc phase, it is determined by spatial orientation with Mn.
(ii) Mn prefers different site occupations in the bcc and fcc phases, which can be understood from the electronic density of states near Fermi energy. This discrepancy implies the possibility of the redistribution of solute Mn in the Fe matrix during the fcc→bcc phase transition, which is favorable for increasing the driving force of transformation.
(iii) Mn alloying tends to destabilize and stabilize the bcc and fcc phases, respectively. With the increase in Mn, the driving force of the fcc→bcc phase transition decreases, in which the stabilization in the fcc phase plays a dominant role. Antiferromagnetism is recognized as the key reason for the enhanced stability of the fcc phase by Mn alloying.

In conclusion, the present work clarifies the impact mechanisms of Mn alloying in the bcc and the fcc phases and the fcc→bcc phase transition in ferroalloys and thus is expected to design advanced ferroalloys.

## Figures and Tables

**Table 1:** **Equilibrium lattice parameter ($a_{0}^{\mathrm{bcc}}$), net magnetization ($M_{\mathrm{Net}}$) and atom-resolved magnetic moments of Fe ($M_{\mathrm{Fe}}$) and Mn ($M_{\mathrm{Mn}}$) for the bcc phase of the ${\mathrm{Fe}}_{16-x}{\mathrm{Mn}}_{x}$ (x= 0, 1 and 2) alloys.** The ground-state structural models of ${\mathrm{Fe}}_{14}{\mathrm{Mn}}_{2}$ are indicated in bold.

Alloy	Model	$a_{0}^{\mathrm{bcc}}$	$M_{\mathrm{Net}}$	$M_{\mathrm{Fe}}$	$M_{\mathrm{Mn}}$	$E_{\mathrm{Total}}$
		**Å**	**µ** ${}_{B}$ **/at.**	**µ** ${}_{B}$ **/at.**	µ${}_{B}$**/at.**	**eV/at.**
Fe	−	2.831	2.19	2.19	−	−8.243
	−	2.84 ${}^{a}$	2.17 ${}^{a}$			
	−	2.83 ${}^{b}$	2.22 ${}^{b}$			
${\mathrm{Fe}}_{15}{\mathrm{Mn}}_{1}$	−	2.824	2.11	2.18	1.05	−8.266
${\mathrm{Fe}}_{14}{\mathrm{Mn}}_{2}$	**I**	**2.829**	**2.02**	**2.23**	**0.46**	**−8.298**
	**II**	**2.820**	**2.02**	**2.17**	**1.02**	**−8.298**
	III	2.819	2.01	2.13	1.06	−8.293
	IV	2.820	2.03	2.07	1.70	−8.286

${}^{a}$ Ref. [43]; ${}^{b}$ Ref. [24].

**Table 2:** **Equilibrium lattice parameter ($a_{0}^{\mathrm{fcc}}$), net magnetization ($M_{\mathrm{Net}}$) and atom-resolved magnetic moments of Fe ($M_{\mathrm{Fe}}$) and Mn ($M_{\mathrm{Mn}}$) for the fcc phase of the ${\mathrm{Fe}}_{16-x}{\mathrm{Mn}}_{x}$ (x= 0, 1 and 2) alloys.** The ground-state structural model of ${\mathrm{Fe}}_{14}{\mathrm{Mn}}_{2}$ is indicated in bold.

Alloy	Model	$a_{0}^{\mathrm{fcc}}$	$M_{\mathrm{Total}}$	$M_{\mathrm{Fe}}$	$M_{\mathrm{Mn}}$	$E_{\mathrm{Total}}$
		**Å**	**µ** ${}_{B}$ **/at.**	**µ** ${}_{B}$ **/at.**	**µ** ${}_{B}$ **/at.**	**eV/at.**
Fe	−	3.478	1.02	1.02	−	−8.095
	−	3.48 ${}^{a}$	1.23 ${}^{a}$			
	−	3.588 ${}^{b}$	0.75 ${}^{b}$			
${\mathrm{Fe}}_{15}{\mathrm{Mn}}_{1}$	−	3.497	0.77	0.98	−2.39	−8.159
${\mathrm{Fe}}_{14}{\mathrm{Mn}}_{2}$	I	3.522	0.93	1.4	−2.33	−8.220
	II	3.507	0.79	1.26	−2.48	−8.225
	III	3.502	0.75	1.23	−2.37	−8.229
	**IV**	**3.512**	**0.83**	**1.34**	**−2.58**	**−8.231**

${}^{a}$ Ref. [24]; ${}^{b}$ Ref. [49].

## Data Availability

The data presented in this study are available on request from the corresponding author.
